# Patients With Hidradenitis Suppurativa Negatively Perceive Both Medical and Euphemistic Appellations of Their Disease: A Study From Turkey

**DOI:** 10.5826/dpc.1104a92

**Published:** 2021-10-01

**Authors:** Gulsen Akoglu, Pelin Esme, Irem Yildiz

**Affiliations:** 1 University of Health Sciences, Gulhane Training and Research Hospital, Department of Dermatology and Venereology, Ankara, Turkey; 2 Hacettepe University Faculty of Medicine, Department of Psychiatry, Ankara, Turkey

**Keywords:** hidradenitis suppurativa, euphemism, folk name, medical appellation, psychodermatology

## Abstract

**Background:**

The use of medical terms and folk names (euphemisms) affect a patient’s understanding of diseases and perceptions of severity.

**Objectives:**

We determine the psychological effects on patients with hidradenitis suppurativa of medical and folk names of their disease.

**Methods:**

This was a cross-sectional and exploratory study conducted at a tertiary referral university hospital in Turkey. A questionnaire on the medical and folk names of hidradenitis suppurativa was administered to 31 males and 25 females.

**Results:**

The patients expressed that they found the medical term hidradenitis suppurativa to be incomprehensible because it is a foreign term. When hearing it for the first time, it evoked negative responses such as confusion and worry about their health. Half of the patients preferred their doctors to use a more understandable and pronounceable name. More than 80% of patients expressed feeling depressed and stigmatized by the folk name of their disease. They preferred the terms boils, abscesses, or hidradenitis when referring to their disease.

**Conclusion:**

Both medical and folk names for hidradenitis suppurativa have negative effects on patients, and most patients feel stigmatized by either term.

## Introduction

During medical consultations, effective communication also requires taking a patient’s social and cultural background into account, as language may influence perception and beliefs in relation to an illness. The use of medical terms and folk names (euphemisms) affect understanding the nature of diseases and perceptions of their severity and importance [1, 2]. For example, the medical term hidradenitis suppurativa (HS), which has ancient Greek roots [3], may sound foreign to a patient whose language does not have similar origins or pronunciations. Additional time may be required to describe the disease in detail.

Euphemisms are substitutions for words or phrases that are used for names or topics that are considered distasteful [4]. People tend to use euphemistic words for upsetting concepts, such as names of diseases and death. For instance, people prefer to use “relentless illness” for cancer or “pass away” for death, and this is because of long-held taboos and taboo-based fears [5]. In ancient Turkey, people believed that a boil or abscess (*ciban* in Turkish) spreads when its name is mentioned. To address this fear, more than 80 euphemistic terms for skin abscesses located in various body regions have evolved [6]. Among these euphemistic words, it is common to use parts of animals’ appellatives resembling the disease features to represent different kinds of boil diseases. For example, due to the resemblance of HS axillary regions to the nipples of the dog, “dog nipple disease” has been used to name HS abscesses located at the level of the armpits [7]. In other countries, people use different names for HS according to their culture or social life. In Spanish-speaking countries, people use *golondrino* meaning “swallow” due to the resemblance of axillary lesions of HS to the nests of swallows. Other commonly used euphemistic expressions, that define HS and related abscesses in familiar and popular contexts, include “cow’s milk-filled nipples” in Russia, “recurrent ingrown hair, recurrent boils, Verneuil’s disease” in France, “rotten armpit” in Afghanistan, and “boils/boil disease” in Canada, United States of America, Azerbaijan, Slovenia, Brussels, and other European countries.

Psychological effects due to the use of medical and/or folk names (euphemisms) for HS are unknown in patients. In daily practice, we observe that patients diagnosed with HS react or comment differently when talking about HS or its corresponding folk expression (euphemism).

## Objectives

In this study, we aimed to determine the psychological effects on HS patients, generated by the choice of medical or folk names to define their disease.

## Methods

This cross-sectional and exploratory study was conducted in the Department of Dermatovenereology in Gulhane Training and Research Hospital, University of Health Sciences in Ankara, Turkey. All consecutive patients with HS, who were diagnosed and followed up in the HS outpatient clinic of our center were invited for the study from July 1 to September 30, 2020. Two illiterate and cognitively impaired patients, unable to read and understand the scales used in the study, together with a patient who was unwilling to participate, were excluded from the study. A total of 56 patients over 18 years were recruited. Patients were examined by the same investigator and their sociodemographic and clinical history and characteristics were recorded. The Hurley staging system was used to evaluate the clinical severity of the disease. Stages range from 1 to 3 as follows: stage 1 (mild), stage 2 (moderate), or stage 3 (severe) [8].

### Informed Consent

Patients who participated in this study gave written informed consent for the publication of their case details.

### Ethics Approval

The local ethics committee provided ethical clearance and approval for this study (Approval ID: 2020-349).

### Interviews and Quality of Life Measurements

Patients were interviewed in a silent and restful room in the same center and were required to fill in a questionnaire including questions regarding the medical and folk names (euphemisms) used to define HS in public contexts. Each interview was recorded by the same investigator and was completed in approximately 20 minutes, after which each patient filled in the scales.

### Interviews on Medical and Folk Expression of Hidradenitis Suppurativa

A questionnaire with 11 and 10 questions concerning the medical and folk (euphemistic word) expressions of HS, respectively, was administered to evaluate thoughts, beliefs, emotions, attitudes, and behaviors of patients toward the names of their disease. Some questions were open-ended, whereas others were close-ended (polar). A pilot test was previously conducted on 10 patients to evaluate and confirm understanding of the questionnaire.

### Statistical Analysis

Statistical analyses were performed using the Statistics Package for the Social Sciences (SPSS) for Windows version 25.0 (IBM, Armonk, NY, USA). Patients’ characteristics are presented as means and standard deviations for continuous variables and as frequencies and proportions for categorical variables. Comparisons between 2 categorical variables were performed using the chi-square analysis. A P value of <0.05 was considered statistically significant.

## Results

Thirty-one males and 25 females (age range: 18–66 years; mean age: 33.5±11.3 years) were enrolled in the study (Table 1). Most of the patients were in clinically severe stages.

Almost all patients first heard hidradenitis suppurativa as the medical term of their disease from their consultant doctor (Table 2). Patients reported finding HS incomprehensible, because it is a foreign term and evokes negative feelings when heard. Only 1 patient with a biology background understood and described the term as an inflammation affecting sweat glands. Although half of the patients preferred hearing the medical expression from their doctor, only 9 patients preferred the name “hidradenitis” for daily life communication. Three patients used the abbreviation “HS” for easy pronunciation. Conversely, 1 patient avoided the use of “HS” as this abbreviation was similar to “MS” (multiple sclerosis), a profoundly serious neurological disease.

Almost 70% of patients were aware of the folk expression “dog nipple disease” mostly learned from a doctor (Table 3). More than 80% of patients expressed negative, depressive, and stigmatizing feelings toward this euphemistic expression. They rather preferred to use boils/abscesses or hidradenitis when talking about their disease in daily life (Table 4).

Both genders expressed discomfort for the use of the expression “dog nipple disease” during discussions with their doctors, close relatives, or other people (all P >0.05). Female patients felt more uncomfortable using this term with their relatives (P <0.05), when compared to male patients in the same context.

Details of the answers to the questionnaire are listed in Tables 2–4.

## Discussion

Hidradenitis suppurativa is a challenging disease and patients are mostly depressed and feel stigmatized [9, 10]. Signs and symptoms of HS reduce the quality of life, affect social lives, and it has a difficult course [11]. The present study shows the different effect produced by the disease different naming. Results show that both medical and euphemistic expressions have negative effects on disease perception and affect patients’ psychosocial life.

Doctors should establish effective communication with their patients during consultation. Physicians might choose medical expressions when communicating, to emphasize the importance and treatment of the disease, especially when the disease is chronic, disabling, or life-threatening and requires that patients and/or their families comply with recommendations and treatments [12]. Medical expressions of diseases may facilitate the contribution of the patient to the problem, decision-making process, and treatment compliance [13], although this is not always the case. For instance, the medical term heart failure has been reported to cause anxiety and fear in patients [1, 2]. Similarly, our data indicate that “HS” evoked confusion and concern in patients because the term sounds weird and difficult to pronounce. As a result, most patients perceived the condition described by the term as serious. Nonetheless, half of the patients preferred adopting the definition provided by their doctors. This is probably due to the confidence patients had in their doctor’s knowledge of the disease and familiarity with the medical expression. Conversely, the other half of the patients preferred referring to a more understandable and pronounceable name during medical consultation. This finding suggests that the medical term “HS”, confounds patients when they first hear the expression during consultation. Patients usually avoid to publicly share the medical term because of the fear of stigmatization, preferring to use alternative definitions.

Although it has progressively become consensual in medical literature, there is a long-lasting controversy regarding the medical naming of HS; some authors claim HS to be a misnomer and suggest using acne inversa as a more proper definition describing the lesions’ histology and intertriginous localizations [14]. On the contrary, although a misnomer, some authors suggest unaltering HS naming, since the disease has several characteristics other than the ones defined by histology [15]. In addition, our findings show that patients also have some problems with HS’ current medical expression.

Various societies and physicians usually tend to use folk names (euphemistic words) to protect patients from the negative effect of bad news and from the loss of hope and perseverance during the disease process [16]. In this study, when patients were asked about the euphemistic expressions of the disease, a significant portion indicated that they were aware of the “dog nipple disease” naming and almost 80% of them heard this folk expression from a clinician (during consultation, on a medical website, or on TV). Although this study was not conducted to investigate the need for clinicians to use euphemisms during conversations on HS, we may speculate on some issues. Euphemisms can help a physician to explain the diagnosis or condition of the disease to the patient by facilitating the understanding of the disease and reducing patient’s stress levels [12]. For example, the use of medical terms, such as obesity, may be perceived as stigmatizing and blamed by the patient [1, 17]. For this reason, clinicians may prefer using euphemisms (your weight may be affecting your health, etc.) to avoid upsetting patients. The tendency to use euphemisms in a medical conversation with HS patients might be totally different and might depend on how patients’ deal with the HS’ difficult definition. Physicians may consider adopting euphemistic terms to describe HS, adapting to the patients’ level of understanding during consultation. Communicating with folk names (euphemisms) supposedly helps patients to figure the disease and effectively engages them in medical conversation.

Although the sample size was not large, the study results showed that using folk names for HS, causes many psychosocial problems. These expressions have significant negative effects on patients, many of whom feel annoyed when they hear them from their doctors. Moreover, they feel uncomfortable using folk names when mentioning the disease with other people or with family members; female patients reported more discomfort than males. These consequences might be due to the sexual connotation perceived from the word “nipple”, which might generate some sense of shame. The stigma seems to have a debilitating effect on patients leading them to choose the disease definition they feel more comfortable with. Patients seem to tolerate the inconvenience caused by the folk expression “dog nipple disease” when adopted by their doctor. This is due to the respect patients have towards physicians’ expertise, authority, and knowledge. This indicates that the euphemistic expression “dog nipple disease” to define HS, is inappropriate in both consultations and public conversations. The term might have been useful and needed during ancient times, however, it is not positively accepted nowadays, as the perception derived from euphemisms’ use has changed. The original word “crippled” well illustrates this kind of change. It evolved from “handicapped” to “disabled,” and then to “people with disabilities” in time, showing how new euphemisms replace previous ones [16]. Similarly, this study indicates that there is a taboo linked to the euphemism “dog nipple disease”. We suggest the use of the medical term “HS” until suitable euphemistic expressions are created.

## Conclusions

The medical and euphemistic expressions for HS have negative effects on patients, and most patients feel stigmatized by their diagnosis when named with either term. Studies on stigmatization and psychological burden of HS should also include analyses on the effects of names attributed to HS, in relation to the population’ social and cultural background.

## Figures and Tables

**Table 1:** Sociodemographic and Clinical Features of Patients

Characteristics	N (%)
Male/Female	31 (55.4)/25 (44.6)
Range, mean ±SD (years)	18–66; 33.5±11.3
18–40 years	41 (73.2)
41–60 years	15 (26.8)
Clinical severity	
Hurley stage 1	14 (25)
Hurley stage 2	14 (25)
Hurley Stage 3	28 (50)
Age at disease onset(years)	13.0–64.5 (25.6±10.0)
Age at the diagnosis of HS (years)	13.0–64.0 (29.2±10.0)
Disease duration(years)	0.0–25.0 (7.9±6.6)
Involved body regions	
Axilla	42 (75.0)
Inguinal	43 (76.8)
Inframammary	7 (12.5)
Gluteal/perianal/intergluteal	22 (39.3)
Other sites/atypical sites	31 (55.4)
Current treatments	
None	9 (16.1)
Topical antibiotics	8 (14.3)
Systemic antibiotics	12 (21.4)
Oral isotretinoin	2 (3.6)
Biologics	25 (44.6)

SD = standard deviation.

**Table 2:** Answers to Questions About the Medical Term Hidradenitis Suppurativa

Questions and Answers	N (%)
**Do you know the medical name of your disease?** (n=56)	
No, I do not know the medical name of my disease	5 (8.9)
Yes, the name is HS	51 (91.1)
**From whom/where did you first hear about HS as the name of your disease?** (n=51)	
From the doctor who examined me	50 (98.1)
From an internet search of my lesions	1(1.9)
**Do you think that the name “HS” is understandable?** (n=56)	
Yes	1 (1.8)
No, because it is a foreign name, not in Turkish 55 (98.2)	
**Additional issues mentioned specifically about the term “HS”** (n=56)	
The term is hard to pronounce	13 (23.2)
It is a weird name	3 (5.4)
It is a complicated term	5 (8.9)
I cannot keep in mind and remember	5 (8.9)
The term is not informative	2 (3.6)
Other people think it is a bad disease	2 (3.6)
Other people think it is a weird name	1 (1.8)
Other people laugh at me when they hear the term	1 (1.8)
I had to search the meaning of the term	1 (1.8)
It makes no sense to me	1 (1.8)
**How did you feel when you heard the name “HS”?** (n=56)	
Puzzled about the name	20 (35.7)
Feared/worried about his/her health	18 (32.1)
Had to search what kind of disease it is on internet	7 (12.5)
Thought it was a bad disease	6 (10.7)
Demoralized	3 (5.4)
Thought I had cancer	2 (3.6)
Relieved to have a diagnosis after 11 years	1 (1.8)
Embarrassed that she could not say the name	1 (1.8)
Nothing, I trust my doctor	7 (12.5)
**Do you prefer your doctor uses another name for your disease instead of using HS?** (n=56)	
Yes, I prefer to hear a more understandable and pronounceable disease name	28 (50.0)
No, I do not	28 (50.0)
I have got used to hearing the medical term; do not want any change	15 (26.8)
I prefer to hear a medical term from the doctor	11 (19.6)

HS = hidradenitis suppurativa.

**Table 3:** Answers to Questions About the Folk Expression “Dog Nipple Disease”

Questions and Answers	N(%)
**Do you know any folk name used in public for your disease?** (n=56)	
Yes, “dog nipple disease”	39 (69.6)
No	17 (30.4)
**From whom/where did you first hear the folk term “dog nipple disease”?** (n=39)	
From a doctor	34 (87.2)
From an internet search of HS/my lesions	19 (48.7)
From the doctor who examined me	14 (35.9)
From a doctor on television	1 (2.6)
From a relative/other person	5 (12.8)
**How did you feel when you learned about the name “dog nipple disease” used for your disease?** (n=39)	
It is disgusting/repulsive/inappropriate/ugly name	8 (20.5)
It is an astonishing term	6 (15.4)
Felt bad when I heard the name	5 (12.8)
It makes sense to use “dog nipple disease,” since it resembles the lesions	4 (10.3)
Felt weird/shame when first heard, then got used to it	3 (7.7)
It is a shameful term	2 (5.1)
The disease does not resemble dog nipples	2 (5.1)
I did not mind	2 (5.1)
It is an insulting name	1 (2.6)
I thought I had a more serious disease than I considered	1 (2.6)
Do not use the name because people make fun of me	1 (2.6)
Mentioning the nipple with disease makes me feel uncomfortable	1 (2.6)
Felt uncomfortable to hear an animal and nipple name in the disease	1 (2.6)
Feared to imagine many nipple-like lesions will spread to every part of the body when the disease progresses	1 (2.6)
The term sounds like an animal disease	1 (2.6)
Felt uncomfortable to think that people imagine dog nipples	1 (2.6)
**Does it annoy you that your doctor calls the name of the disease as “dog nipple disease”?** (n=39)	
Yes	22 (56.4)
No	17 (43.6)
**When talking to your relatives, do you feel uncomfortable saying the name of your disease as dog nipple disease?** (n=39)	
Yes	23 (59.0)
No	16 (41.0)
**When talking to other people, do you feel uncomfortable saying the name of your disease as dog nipple disease?**	
Yes	33 (84.9)
No	6 (15.4)

HS = hidradenitis suppurativa.

**Table 4:** Preferred Expressions Patients Use to Define Their Lesions/Disease (n=56)

Expressions	N (%)
Boils/abscess	12 (21.4)
Hidradenitis	9 (16.1)
Pus	8 (14.3)
Acne	5 (8.9)
Hair root pus	4 (7.1)
HS (abbreviation)	3 (5.4)
Dog nipple disease	3 (5.4)
Wound	2 (3.6)
Ingrown hair	1 (1.8)
Fungal disease	1 (1.8)
Not defined	8 (14.3)
